# Brain age as a biomarker for pathological versus healthy ageing – a REMEMBER study

**DOI:** 10.1186/s13195-024-01491-y

**Published:** 2024-06-14

**Authors:** Mandy M.J. Wittens, Stijn Denissen, Diana M. Sima, Erik Fransen, Ellis Niemantsverdriet, Christine Bastin, Florence Benoit, Bruno Bergmans, Jean-Christophe Bier, Peter Paul de Deyn, Olivier Deryck, Bernard Hanseeuw, Adrian Ivanoiu, Gaëtane Picard, Annemie Ribbens, Eric Salmon, Kurt Segers, Anne Sieben, Hanne Struyfs, Evert Thiery, Jos Tournoy, Anne-Marie van Binst, Jan Versijpt, Dirk Smeets, Maria Bjerke, Guy Nagels, Sebastiaan Engelborghs

**Affiliations:** 1 Department of Biomedical Sciences, University of Antwerp, Antwerp, Belgium; 2 Department of Neurology, Universitair Ziekenhuis Brussel (UZ Brussel), Brussels, Belgium; 3 Neuroprotection and Neuromodulation (NEUR) Research Group, Center for Neurosciences (C4N), Vrije, Universiteit Brussel (VUB), Brussels, Belgium; 4 icometrix, Leuven, Belgium; 5 Centre of Medical Genetics, University of Antwerp, and Antwerp University Hospital - UZA, Edegem, Belgium; 6 GIGA-CRC-IVI, Liège University, Allée du Six Août, 8, Liège, 4000 Belgium; 7 Geriatrics Department, Brugmann University Hospital, Universite Libre de Bruxelles, Brussels, Belgium; 8 Neurology Department, AZ St-Jan Brugge, Brugge, Belgium; 9 Ghent University Hospital, Ghent, Belgium; 10 Neurological department H. U. B. - Erasme Hospital – Vrije Universiteit Brussel (VUB), Brussels, Belgium; 11 Laboratory of Neurochemistry and Behavior, Experimental Neurobiology Unit, University of Antwerp, Antwerp, 2610 Belgium; 12 Memory Clinic, Ziekenhuisnetwerk, Antwerp, Belgium; 13 Institute of Neuroscience, Université Catholique de Louvain, Brussels, 1200 Belgium; 14 Department of Neurology, Clinique Universitaires Saint-Luc, Brussels, 1200 Belgium; 15 WELBIO Department, WEL Research Institute, Wavre, 1300 Belgium; 16 Department of Neurology, Cliniques Universitaires St Luc, and Institute of Neuroscience, Université Catholique de Louvain, Woluwe-Saint-Lambert (Brussels), Belgium; 17 Department of Neurology, Clinique Saint-Pierre, Ottignies, Belgium; 18 Department of Neurology, Memory Clinic, Centre Hospitalier Universitaire (CHU) Liège, Liège, Belgium; 19 Memory Clinic - Neurology and Geriatrics Department, CHU Brugmann, Van Gehuchtenplein 4, Brussels, 1020 Belgium; 20 Neuropathology Lab, IBB-NeuroBiobank BB190113, Born Bunge Institute, Antwerp, Belgium; 21 Department of Pathology, Antwerp University Hospital - UZA, Antwerp, Belgium; 22 Laboratory of Neurology, Translational Neurosciences, Faculty of Medicine and Health Sciences, University of Antwerp, Antwerp, Belgium; 23 Johnson and Johnson Innovative Medicine, Beerse, Belgium; 24 Department of Neurology, University Hospital Ghent, Ghent University, Ghent, Belgium; 25 Department of Chronic Diseases, Metabolism and Ageing, Geriatric Medicine and Memory Clinic, University Hospitals Leuven and KU Leuven, Louvain, Belgium; 26 Radiology Department, Universitair Ziekenhuis Brussel (UZ Brussel), Brussels, Belgium; 27 Department of Clinical Chemistry, Laboratory of Neurochemistry, Universitair Ziekenhuis Brussel (UZ Brussel), Brussels, Belgium; 28 St. Edmund Hall, University of Oxford, Oxford, UK; 29 AIMS lab, Center for Neurosciences (C4N), Vrije Universiteit Brussel, UZ Brussel, Brussels, Belgium

**Keywords:** Alzheimer’s disease, Magnetic resonance imaging, Biomarker, Brain predicted age difference, Brain age, Automated volumetry

## Abstract

**Objectives:**

This study aimed to evaluate the potential clinical value of a new brain age prediction model as a single interpretable variable representing the condition of our brain. Among many clinical use cases, brain age could be a novel outcome measure to assess the preventive effect of life-style interventions.

**Methods:**

The REMEMBER study population (*N* = 742) consisted of cognitively healthy (HC,*N* = 91), subjective cognitive decline (SCD,*N* = 65), mild cognitive impairment (MCI,*N* = 319) and AD dementia (ADD,*N* = 267) subjects. Automated brain volumetry of global, cortical, and subcortical brain structures computed by the CE-labeled and FDA-cleared software icobrain dm (dementia) was retrospectively extracted from T1-weighted MRI sequences that were acquired during clinical routine at participating memory clinics from the Belgian Dementia Council. The volumetric features, along with sex, were combined into a weighted sum using a linear model, and were used to predict ‘brain age’ and ‘brain predicted age difference’ (BPAD = brain age–chronological age) for every subject.

**Results:**

MCI and ADD patients showed an increased brain age compared to their chronological age. Overall, brain age outperformed BPAD and chronological age in terms of classification accuracy across the AD spectrum. There was a weak-to-moderate correlation between total MMSE score and both brain age (*r* = -0.38,*p* < .001) and BPAD (*r* = -0.26,*p* < .001). Noticeable trends, but no significant correlations, were found between BPAD and incidence of conversion from MCI to ADD, nor between BPAD and conversion time from MCI to ADD. BPAD was increased in heavy alcohol drinkers compared to non-/sporadic (*p* = .014) and moderate (*p* = .040) drinkers.

**Conclusions:**

Brain age and associated BPAD have the potential to serve as indicators for, and to evaluate the impact of lifestyle modifications or interventions on, brain health.

**Supplementary Information:**

The online version contains supplementary material available at 10.1186/s13195-024-01491-y.

## Introduction

Despite advancements in research toward anti-amyloid (Aβ) therapies, which have shown potential in reducing Aβ brain accumulation, their effectiveness in improving cognition in late-stage clinical trials has been minimal, prompting reconsideration of the magnitude of the role of Aβ in Alzheimer’s disease (AD) pathogenesis [1]. Since AD up to today remains without a cure, additional focus is being put on prevention strategies, including the promotion of healthy behaviors. Throughout the years, improvement in quality of life has notably aided in a prolonged preservation of cognitive and mental functioning and overall brain integrity, and vice versa [2]. However, in our growing and ageing population, even though the age-specific incidence of dementia seems to be falling [3], the prevalence of dementia is still rising.

Brain health is more than the mere absence of disease or impairment. While chronological age is the main risk factor of dementia [4], several studies have shown that age and neurodegeneration do not follow the same trajectory [5, 6]. There is a noticeable variability in the onset and progression of neurodegenerative alterations among individuals, with some experiencing earlier onset or faster progression, while others potentially having a slower or delayed decline in neurological function compared to persons of the same age group. Factors that could shape our cognitive reserve and influence the development of dementia are numerous, including blood pressure, education, hearing capacity, smoking behavior, alcohol abuse, presence of depression, diabetes, and obesity, traumatic brain injury, as well as (a lack of) physical activity and social engagement [3, 7]. Their potential use as targets for prevention strategies is increasingly being investigated, to determine if and how lifestyle (changes) might slow down or prevent progression to dementia. This window of opportunity motivates the development of a novel target, namely an objective and comprehensible measure of brain health.

In an endeavor to frame this complex concept of brain health, machine learning methods [8–11] have been proposed to express brain health in terms of brain ageing. These models estimate an individual’s ‘brain age’ based on the structural variations and alterations in different regions of the brain, which can be interpreted as a neuroimaging-driven marker of brain health. As brain age is sensitive to lifestyle- and health-related factors [12], it could serve as a (secondary) reliable objective metric, either parallel to other measures of ageing, such as physiological (incl. ECGs, EEGs, imaging for tumor detection and lesion identification, blood tests (e.g. inflammation markers, vitamin B12, cholesterol levels etc.)) and neuropsychological examinations, or separately in case these other measures are not available. It is important to recognize that current physiological and neuropsychological examinations are not designed, nor sufficiently equipped, to objectively and consistently capture the multifaceted influence of lifestyle and health-related factors on their determined outcomes.

A brain age estimation higher than that of healthy age-matched peers has already been linked to AD [13–16], Parkinson’s disease [17], Schizophrenia [16, 18–20], Multiple Sclerosis [21–23], as well as to life expectancy [24]. Various brain age models exist [25], but tend to be rather complex in their interpretation. In relation to AD brain age research, previous literature mainly focused on publicly available neuroimaging datasets [14, 15, 26–28], symptomatic AD [8, 13, 14, 29–31], had limited generalizability of findings to more diverse populations due to population bias [14, 32, 33], were constricted to the use of 1.5T MRI scans [34], or exclusively conducted cross-sectional analysis [28]. In addition, there is a notable scarcity of studies exploring BPAD using a longitudinal approach [35, 36] and if conducted in a real-world clinical context, sample sizes are small [37]. Consequently, there is an underrepresentation of (longitudinal) brain-age studies that are representative of the general population at-risk for dementia.

In this paper, the main and first objective is to clinically evaluate a new brain age model on real-world clinical data and MRI scans of subjects across the entire AD continuum, as a step towards clinical use of a single interpretable variable representing the condition of our brain. The model, characterized by its linear design, is based on volumetric assessment by a highly accurate brain segmentation software designed for the assessment of clinical MRI scans, and was validated previously on a multiple sclerosis cohort [21].

Furthermore, considering the added value to test for effectiveness of lifestyle changes / interventions on brain health and thus prevention of dementia [16], the second objective is to investigate whether the difference between chronological and brain age, i.e., brain-predicted age difference (BPAD = brain age – chronological age), is associated with indicators of disease incidence and progression such as the time to conversion from MCI to dementia due to AD (ADD) and MMSE decline. Finally, the effect of several lifestyle factors on BPAD will be evaluated. Our ultimate aim is to provide insights into the complexity of brain age trajectory and its interpretation in real-world clinical settings, addressing the challenges and nuances involved in understanding brain aging patterns across different stages of Alzheimer's disease.

## Methods

### Study population and design

The study population (*N* = 742) consisted of a subset of the ‘retrospective Belgian multi-center MRI biomarker study in dementia’ (REMEMBER, *N* = 887) that underwent a baseline (BL) brain MRI scan, as well as a clinical neurological and neuropsychological examination [38]. Participants were recruited from eight different memory clinics that are members of the Belgian Dementia Council (BeDeCo). Patients were classified in compliance with the National Institute on Aging and Alzheimer's Association (NIA-AA) criteria for ‘MCI due to AD’ and ‘Dementia due to AD’ [39–42]. Subjective cognitive decline (SCD) patients were diagnosed according to the criteria of Jessen et al. (2014) [43, 44]. Cognitively healthy controls (HC) were selected among available (research) cohorts, such as spouses of patients who visited the memory clinic and community-dwelling volunteers. These subjects underwent at least a cognitive screening test to exclude cognitive impairment and were required not to meet the criteria for SCD as formulated by Jessen et al. (2014) [43, 44]. The level of education for each participant was defined as the number of years of school completed. Obtaining a diploma was not a requirement. The study was approved by the ethics committee of University of Antwerp / Universitair Ziekenhuis Antwerpen, Antwerp (N°16/2/18) and by the ethics committees of Algemeen Ziekenhuis Sint-Jan Brugge-Oostende, Brugge (N°1992); Centre Hospitalier Universitaire Brugmann (CHU Brugmann), Brussels (N°2016/84); Centre Hospitalier Universitaire Liège (CHU Liège), Liège (N°2012/274); Cliniques Universitaires de Bruxelles (ULB), Hôpital Erasme, Brussels (N°P2016/187); Cliniques Universitaires Saint-Luc (UCL), Brussels (N°2016/07jui/261); Cliniques St-Pierre Ottignies, Ottignies (N°OM045); Universitair Ziekenhuis Brussel, Brussels (N°2016/183); and Ziekenhuis Netwerk Antwerp (ZNA), Antwerp (N°4730).

A subset of the population underwent one or more recurrent brain MRI examinations, intermittently including neuropsychological assessments. Within this subset, baseline and the first follow-up evaluation were equally spaced in time (± 3 months) and comprised MRI examinations and MMSE scores. Subsequent follow-ups, encompassed, at a minimum and if applicable, the occurrence and time to conversion to a later stage in the AD continuum.

For additional information regarding the study population, we refer to Niemantsverdriet et al. [38] and Wittens et al. [45].

### Image acquisition and analysis

T1-weighted (T1w) MRI sequences from the radiology departments of the participating memory clinics were available for each subject. MRI systems used were GE medical systems (1.5T and 3.0T), Philips (1.5T and 3.0T), and Siemens (1.5T and 3.0T)). The following global, cortical and subcortical brain volumes were extracted the CE-labeled and FDA-cleared automated brain volumetry software icobrain dm (dementia) (v. 4.4): gray matter (GM), white matter (WM), frontal (FC), parietal (PC), temporal (TC) and occipital (OC) cortical gray matter, hippocampi (left and right), thalamic (left and right), and lateral ventricles. In brief, after skull stripping and bias field correction, the T1w image was segmented into GM, WM, and cerebrospinal fluid (CSF). After this initial segmentation, an assembly of cortical labels available in Montreal Neurological Institute (MNI) space, a database of manually annotated brain MRI scans, provided the base for sub-segmentation into cortical lobes, thalamic and hippocampal volumes, which have been thoroughly described elsewhere [38, 45, 46]. All computed brain volumes were normalized by intracranial volume to correct for head size.

### Brain age model

To obtain brain age, a model described by Denissen et al., 2022 [21] was used. Concisely, this model was trained on a large dataset of 1673 cognitively healthy controls (age range [min–max]: [18–94] years old, for further details regarding the training dataset, cfr. Denissen et al., 2022.) to predict chronological age from the extracted brain structure volumes and sex using ordinary least squares regression. This resulted in a linear model in which the input features were combined into a weighted sum to yield a brain structure volume-based prediction of the chronological age of a subject, referred to as brain age. The set of weights in this model can then be used to determine the brain age of any individual for whom icobrain dm 4.4 volume measurements and sex are available. The model is easily interpretable due to its linear nature and transforms a complex set of variables to a single easily interpretable metric of brain health, that enables easy interpretation for patients, caregivers, and medical professionals. Given that the brain age model was trained exclusively on healthy controls, which is the convention in brain age research, it was readily applicable for our purposes, without any need for further pathological condition-specific adjustments.

Brain age might deviate from the chronological age of a subject. To quantify this, the BPAD was additionally calculated by subtracting the chronological age from the brain age according to the following formula:

$${BPAD}_{i}= {BA}_{i}- {CA}_{i}$$

where ${BA}_{i}$ represents brain age and chronological is represented as ${CA}_{i}$.

### Statistical analysis

#### Study population demographics

All statistical analyses were performed using the R-environment (R-Studio, v.4.3.1) for statistical computing and graphics using the following “packages” and (functions) [47]. Variable distribution normality was evaluated and confirmed using the Shapiro Wilk normality test using “stats” (shapiro.test), quantile–quantile plots using “ggplot” (ggqqplot) and histograms using “graphics” (hist), in combination with “stats” (resid). Study population demographics were described using mean and standard deviation (SD), percentages (%), and (if data for a specific variable is only available for a subset of the study population) numbers of subjects (N) (R package: “arsenal” (tableby and write2word)) [48]. Distribution of categorical variables within subject groups were analyzed by Chi-square tests. For extracted brain structure volumes and other numeric variables, ANCOVA tests were used. A post-hoc analysis was performed to explore significant differences between groups using Tukey correction (significance level of 0.05), due to its ability to effectively control the family-wise-error rate (adjusting the *p*-value for multiple comparisons) while simultaneously provide pairwise comparisons between groups (R packages: “stats” (lm, anova, chisq.test) [49] and “multcomp” (ghlt)) [50].

#### Cross-sectional analysis

##### Brain age model generalizability

To establish generalizability of the brain age model, the mean absolute error (MAE) between brain age and chronological age was calculated for each diagnostic group (HC, SCD, MCI, and ADD) (see section 'Study population and design' for specifications) (R package: “Metrics” (mae)) [51]. It is computed using the following formula:

$$MAE= \frac{{\sum }_{i=1}^{n}|{BPAD}_{i} |}{n}$$

where MAE stands for mean absolute error, and $\text{BPAD}$​ represent the brain predicted age difference of the ${\text{i}}^{\text{th}}$ individual, respectively. Note that in brain age research, it is customary to report MAE on an unseen cognitively healthy control test set to gauge model performance and allow comparison with other brain age models.

Conversely, evaluating BPAD on this healthy dataset serves as a ‘sanity check’, as it is expected to yield a distribution centered around zero, indicating alignment between brain age and chronological age. After evaluating the model's performance on cognitively healthy data, it can be extended to a pathological sample, where BPAD is typically assessed, as the MAE does not provide a reliable estimate of BPAD distribution variance; typically, the mean BPAD is positive in pathological samples. Thus, unlike BPAD, the MAE considers the absolute value of the difference between chronological and brain age, serving as a statistical performance metric, whereas BPAD can be thought of as a patient-specific biomarker.

Lastly, one-sample t-tests were conducted to assess if the mean value of BPD in HC, SCD, MCI, and ADD groups significantly differs from zero (significance level = 0.05).

##### Correlation analysis

To investigate the relationship between BPAD, brain age, MMSE scores and chronological age, we used the “ggpubr” (ggscatter, ggqqplot) package [52], “stats” (shapiro.test, cor.test) package [49] and the “base” (cor) package. A Pearson's correlation matrix was constructed using the R package 'GGally' (ggpairs) to examine the relationships among multiple variables, including BPAD, brain age, MMSE, and chronological age (R package: “GGally” (ggpairs)) [53]. Each cell in the matrix represents the correlation coefficient between two variables, allowing for a comprehensive assessment of their interrelationships. In addition, the Spearman correlation between BPAD, the annualized BPAD difference and cognitive state over time, expressed as the change on MMSE total score per year, was investigated (FU = follow-up, in years):

$$cognitive\ evolution\ over\ time =\frac{{(MMSE}_{BL}-{MMSE}_{FU})}{{FU}_{years}}$$

##### Classification performance

To test the performance of brain age and BPAD in predicting disease stage, logistic regression was used, with brain age, BPAD and chronological age separately as predictors. The following pairwise combinations of disease stages were considered as binary outcomes: SCD vs. HC, MCI vs. HC, ADD vs. HC, MCI vs. SCD, ADD vs. SCD, and ADD vs. MCI. Classification performance was evaluated using receiver operating characteristic (ROC) analysis, with the R package “pROC” (roc, auc, coords, ci) [54] and the “stats” (predict, glm) package [49]. Area under the curve (AUC), sensitivity and specificity were documented for each pairwise combination of disease stages. Sensitivity and specificity were computed at the optimal cut-off point associated with the Youden index [55] which maximizes the sum between sensitivity and specificity on the ROC curve. ANOVA with post-hoc Tukey correction was conducted to compare the AUC values of the three different predictors (chronological age, BPAD, and brain age), as well as for each pairwise comparison between diagnostic groups. Since they are commonly used to compare AUC values of ROC curves, for each binary classification, DeLong tests [56] were used to additionally confirm if the AUC values between the three variables (by means of pairwise comparisons) were significantly different.

#### Longitudinal analysis

In brain age research, the BPAD variable is typically used for further analyses as it reflects the disease-specific component of brain age, independent of the chronological age of a subject. For the same reason, BPAD was used here to study how pathology impacts cognitive evolution and conversion to more advanced stages across the ADD spectrum. As it is ultimately of interest how modifiable factors such as lifestyle changes could impact disease-specific brain damage, the longitudinal analyses were focused on BPAD, as biological but not chronological aging could be impacted by these interventions.

##### Trajectory analysis

To investigate the changes in brain age and BPAD over time and to see if BPAD is a linear phenomenon, an exploratory longitudinal analysis was conducted on a subset of the study population with longitudinal data containing BL and FU MRI acquisitions. A paired t-test with a significance level of 0.05 was employed to compare brain age and BPAD between BL and FU within the different diagnostic groups. A linear mixed model was fitted to investigate significant differences in change over time between the following pairwise comparisons (baseline diagnosis): SCD vs. HC, MCI vs. HC, ADD vs. HC, MCI vs. SCD, ADD vs. SCD, ADD vs. MCI. BPAD and brain age were investigated separately as dependent outcome variables, using the time between BL and FU MRI and diagnosis including their interaction term as fixed effects (to specifically examine the influence of time and diagnostic groups on the outcome variables), while including subjects as random effects (to account for individual variability within subjects over time). The data was represented as mean ± SD, or percentages, where applicable. Significance of the fixed effects was tested using an F-test with Kenward Roger correction [57] for degrees of freedom.

##### Occurrence of conversion

To analyze the effect of BPAD on the *occurrence* of conversion from MCI to ADD, a logistic regression model was fitted where BPAD was entered as an independent variable, assuming a continuous increase of change from lowest to highest BPAD (R packages: “pROC” (roc, auc, coords, ci) [54] and “stats” (predict, glm) [49]). The occurrence of conversion was treated as a binary variable with two possible outcomes (yes/no).

##### Time to conversion

In addition to the occurrence of conversion, the potential impact of BPAD on the conversion time, specifically exploring whether a higher BPAD results in a faster conversion time, was investigated. Subsequently, the conversion time was plotted against the BPAD using the Kaplan–Meier estimator [58] (R packages: “survminer” and “survival” (survobject, survfit, ggsurvplot)). The Kaplan–Meier estimator calculates survival probabilities using observed event times. It operates on a "time-to-event" model, where the endpoint—specifically, reaching a certain BPAD threshold—predicts the time until a particular event occurs. In this context, the event signifies 'the conversion to one of the later stages in the AD continuum'. Different BPAD thresholds (> 5, > 10, > 15, > 20, > 25, > 30, > 50) were included. The FU time was set to a maximum of 5 years, based on the period of available follow-up data on conversion, indicating that after an absence of conversion in these 5 years, this will be set equivalent to ‘not converted’. The log-rank test was used to compare the survival curves between the aforementioned BPAD thresholds, testing the null hypothesis that two groups do not differ.

#### BPAD and lifestyle factors

To provide insight into the relationship between lifestyle factors and their potential impact on BPAD and brain age, the BPAD and brain age (ANCOVA corrected for age and education, followed by post-hoc analysis with Tukey correction) between different subgroups were compared based on the following two variables: smoking behavior and alcohol usage.

## Results

### Study population

The final study population consisted of cognitively healthy controls (*N* = 91), subjective cognitive decline (*N* = 65), mild cognitive impairment (*N* = 319) and Alzheimer disease dementia (*N* = 267), resulting in a total of 742 participants. Study population demographics and brain structure volumes computed by icobrain dm are presented in Table 1.

**Table 1:** Study population demographics demographics and brain structure volumes

	**HC (*N*** **= 91)**	**SCD (*N*** **= 65)**	**MCI (*N*** **= 319)**	**ADD (*N*** **= 267)**	**Total (*N*** **= 742)**	*p* **value**
**Sex (#,%F)**						0.011^$^
F	49 (53.8%)	32 (48.5%)	156 (48.9%)	166 (62.2%)	403 (54.3%)	
**Chronological Age (years)**						< 0.001
Mean (SD)	67.3 (8.6)^#	68.5 (9.7)^#	74.8 (7.7)* + #	77.6 (8.0)* + ^	74.3 (8.9)	
**Brain Age (years)**						< 0.001
Mean (SD)	69.7(14.9)^#	73.4(19.8)^#	87.6b(18.7)* + #	92.5 (25.1)* + ^	85.9 (22.4)	
**BPAD**						< 0.001
Mean (SD)	2.4 (11.1)^#	5.3 (16.5)^#	12.8 (18.1)* + #	14.9 (25.0)* + ^	11.6 (20.6)	
**MMSE score**						< 0.001
N	71	59	302	259	691	
Mean (SD)	29 (1)	28 (2)	25 (3)	21 (5)	24 (5)	
**Years of education (YOE)**						< 0.001
N	58	58	292	236	644	
Mean (SD)	14.9 (3.8)	14.0 (3.2)	12.8 (4.0)	10.9 (3.9)	12.4 (4.1)	
**WM (mL)**						0.004
Mean (SD)	626 (58)	628 (56)	604 (69)	603 (81)	608 (72)	
**GM (mL)**						< 0.001
Mean (SD)	853 (44)^#	830 (60)#	799 (66)*#	780 (74)* + ^	801 (70)	
**Frontal CGM (mL)**						< 0.001
Mean (SD)	216 (17)^#	213 (24)#	199 (26)*	194 (28)* +	200 (27)	
**Parietal CGM (mL)**						< 0.001
Mean (SD)	140 (13) + ^#	133 (15)*#	126 (16)*#	120 (20)* + ^	126 (18)	
**Occipital CGM (mL)**						0.009
Mean (SD)	63 (11) + ^#	57 (8)*	59 (12)*	58 (13)*	59 (12)	
**Temporal CGM (mL)**						< 0.001
Mean (SD)	150 (10)^#	147 (16)^#	136 (15)* + #	130 (16)* + ^	136 (16)	
**Hippocampus, Left (mL)**						< 0.001
Mean (SD)	4.5 (0.4)^#	4.3 (0.5)*^#	3.9 (0.6)* + #	3.6 (0.7)* + ^	3.9 (0.7)	
**Hippocampus, Right (mL)**						< 0.001
Mean (SD)	4.7 (0.4)^#	4.4 (0.5)*^#	4.1 (0.6)* + #	3.7 (0.8)* + ^	4.0 (0.7)	
**Thalamus, Left (mL)**						< 0.001
Mean (SD)	8.1 (0.6)#	8.1 (0.7)	7.7 (0.6)	7.6 (0.8)*	7.7 (0.7)	
**Thalamus, Right (mL)**						< 0.001
Mean (SD)	8.2 (0.7)^#	8.0 (0.8)	7.7 (0.7)*	7.7 (0.7)*	7.8 (0.7)	
**Lateral Ventricles (mL)**						< 0.001
Mean (SD)	43 (22)^#	48 (23)^#	66 (30)* + #	77 (33)* + ^	65 (32)	

Data represented as mean, standard deviation (SD). Analysis: Chi-square test (categorical variables “^$^”; sex), ANCOVA (corrected for age and education) and *Post-hoc* analysis with Tukey correction (significance between disease stages and MMSE score) Disease stages: Cognitively healthy controls; HC. Subjective cognitive decline subjects; SCD. Mild cognitive impairment patients; MCI. Alzheimer disease dementia patients; ADD. Discrete variables: Mini Mental-State Examination score; MMSE. Continuous variables. Brain age predicted difference; BPAD. White matter; WM. Gray matter; GM. Cortical gray matter; CGM; Baseline, BL. Significant differences indicated by: disease vs. HC “*”; vs. SCD “ + ”; vs. MCI “^”; vs. ADD “#”

### Brain age model generalizability

#### Brain age estimates

**Fig. 1:**
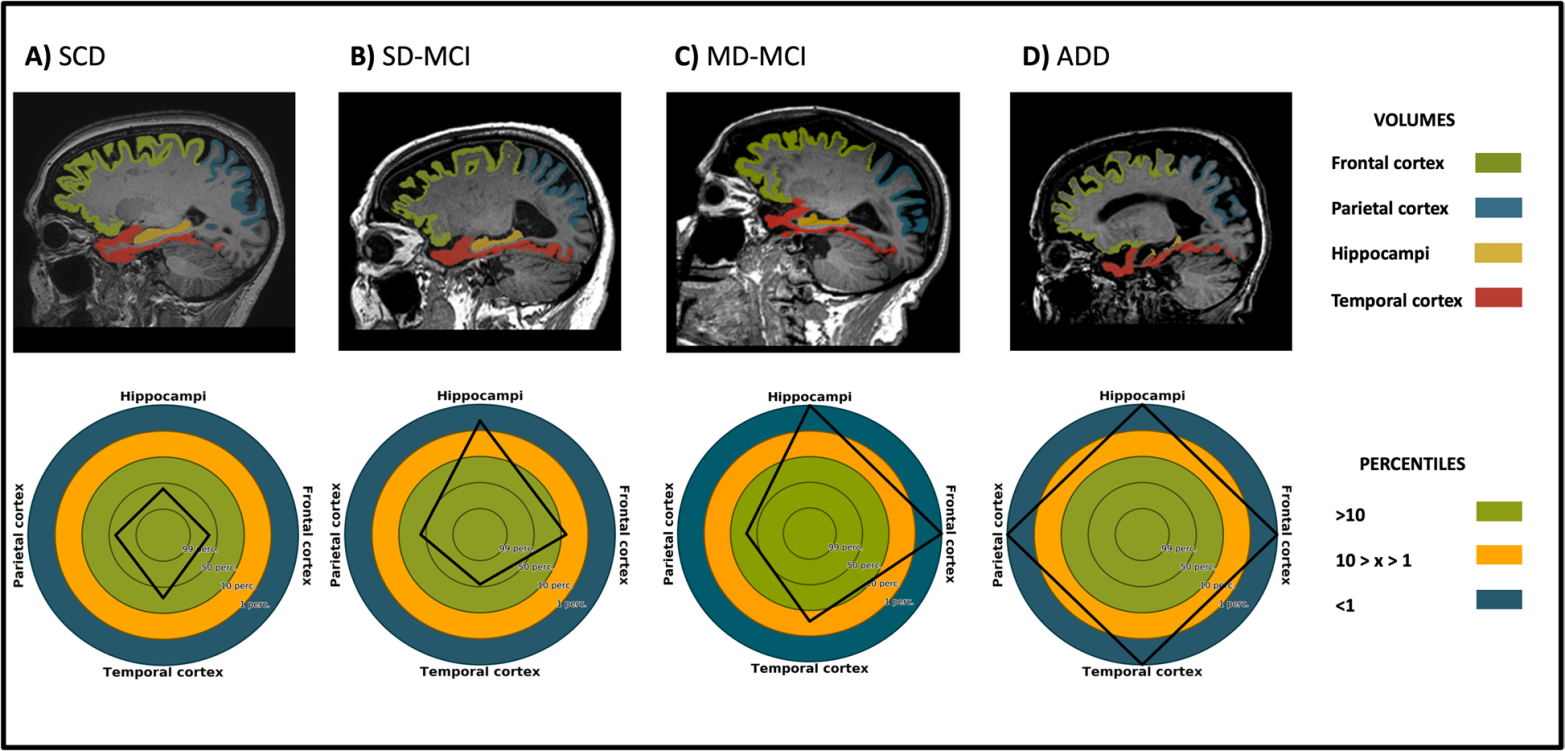
Brain age estimates in three selected cases. Color-coded brain region segmentations based on icobrain dm; Frontal cortex, FC (green), Parietal cortex, PC (blue), Temporal Cortex, TC (red), Hippocampus, HIP (yellow). Volumetric signature circles (below) demonstrate the prevalence of individual patient volumes relative to an age and sex-matched healthy reference population. A value within the green inner circle aligns with volumes observed in > 10% of the reference population, indicating it falls within the normal range. The orange middle circle denotes threshold values that warrant caution and vigilance, corresponding to 10 > x > 1% of the reference population. A value residing within the blue circle signifies a volume found in < 1%, suggesting abnormality. **A** Subjective cognitive decline (SCD) subject with a chronological age of 71.6 years old and a brain age of 60.8 years old, accompanied by below volumetric signature indicating normal FC, PC, TC, and HIP volumes. **B** Single-domain (SD) mild cognitive impairment (MCI) patient with a chronological age of 81.5 years old and a brain age of 89.6 years old, accompanied by below volumetric signature indicating low HIP volume but normal FC, PC, TC, volumes. **C** Multi-domain (MD) MCI patient with a chronological age of 83.3 years old and a brain age of 104.1 years old, accompanied by below volumetric signature indicating low HIP volume and FC volumes, threshold TC volumes, and normal PC volumes **D** Alzheimer’s disease dementia (ADD) patient with a chronological age of 80.9 years old and a brain age of 131.3 years old, accompanied by below volumetric signature indicating generalized cortical atrophy

Visualization of different brain age estimates are depicted together with their volumetric signatures in Fig. 1. The volumetric signature circles compare individual patient volumes to a healthy reference population. Green indicates volumes in over 10% of the population (normal), orange highlights volumes in 1% to 10% (caution), and blue signifies volumes in less than 1% (potential abnormality). Subjects suitable as representatives for low (brain age: 60.8, chronological age: 71.6, SCD subject), intermediate (brain age: 89.6, chronological age: 81.5, Single-domain (SD) MCI patient), intermediate-high (brain age: 104.1, chronological age: 83.3, Multi-domain (MD) MCI patient), and high (brain age: 131.3, chronological age: 80.9, ADD patient) brain age estimations compared to their chronological age were chosen based on MRI scan quality and at this point arbitrarily defined definitions of BPAD thresholds (low brain age: BPAD ≤ 5; low-intermediate: 5 < BPAD < 10; intermediate-high: 10 ≤ BPAD ≤ 30, high: BPAD > 30). No atrophy is seen when looking at the SCD subject with a ‘low’ brain age visualization, while slight frontal and hippocampal atrophy was visually detected, and reported as abnormal hippocampal volumes by the volumetric signature, for the ‘low-intermediate’ brain age stage of the SD MCI patient. The ‘intermediate-high’ brain age MD MCI patient had abnormal hippocampal and frontal cortex volumes. Lastly, atrophy in all color-coded brain regions was seen for the ‘high’ brain age estimation of the ADD patient.

The model predicted brain age with a mean absolute error (MAE) of 8.77 years for cognitively healthy controls, 13.07 years for SCD subjects, 17.09 years for MCI patients, and 20.12 years for ADD patients, respectively. It's important to note that as individuals progress further along the AD continuum, a higher brain age compared to their chronological age can be expected. Therefore, the interpretation of model performance on these groups needs to be made with caution, as it would naturally result in larger errors.

### Brain ageing across the AD spectrum

#### Analyzing BPAD and brain age differences between disease stages

BPAD and brain age differences amongst diagnostic groups (HC, SCD, MCI and ADD) are presented in Table 1. The results show a clear trend, indicating that subjects in more advanced stages of the AD spectrum exhibit higher levels of both brain age and BPAD compared to earlier stages. After correcting for age, significant differences were observed between the following groups for both variables: MCI vs. HC (*p* < 0.001), ADD vs. HC (*p* < 0.001), MCI vs. SCD (*p* < 0.001), ADD vs. SCD (*p* < 0.001), and ADD vs MCI (*p* < 0.049).

**Fig. 2:**
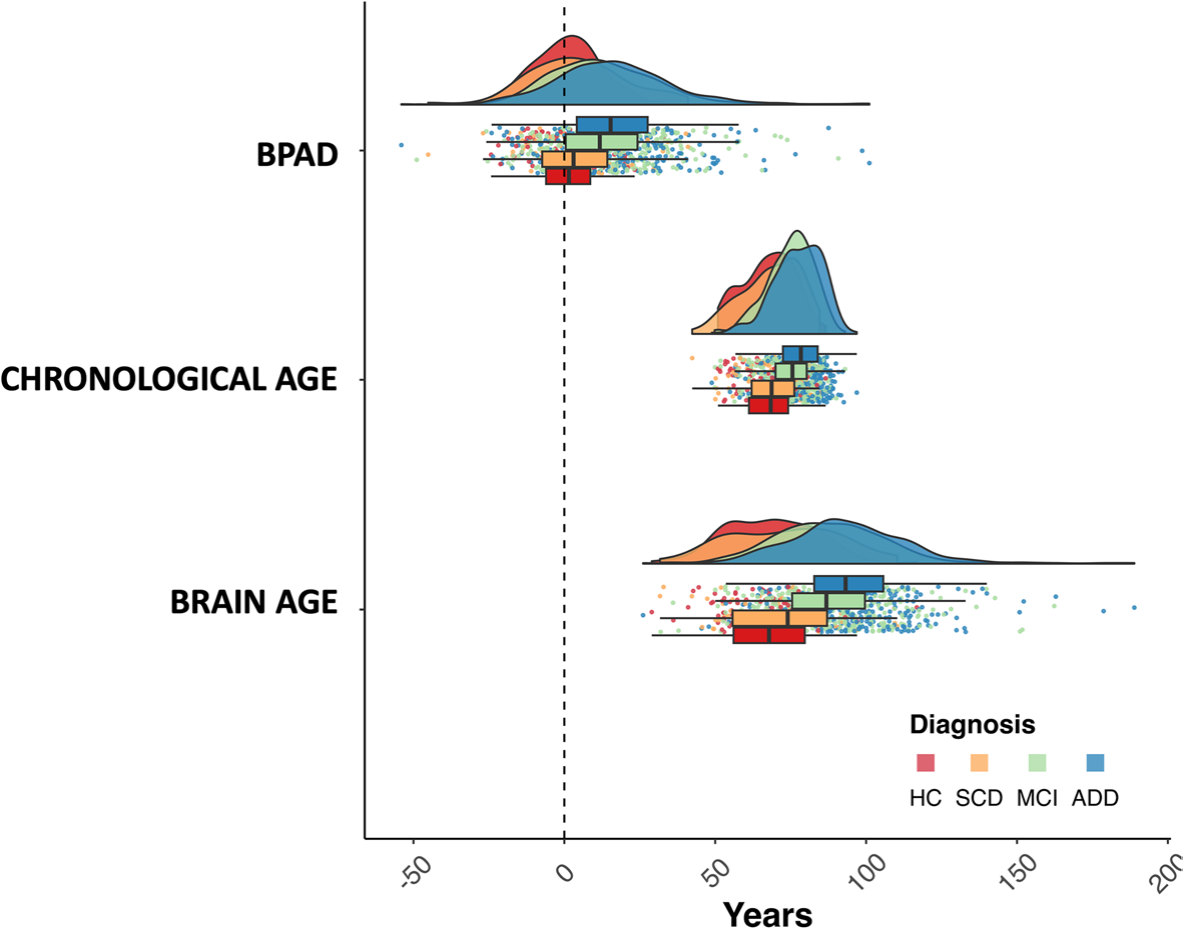
Distribution of age variables per diagnostic group. Cognitively healthy controls; HC. Subjective cognitive decline subjects; SCD. Mild cognitive impairment patients; MCI. Alzheimer disease dementia patients; ADD. Brain predicted age difference; BPAD. Age at baseline; Chronological age. All diagnostic groups are visualized (HC in red, SCD in orange, MCI in green, and ADD in blue)

When visually comparing BPAD and brain age distributions to chronological age, MCI and ADD patients confirmed a higher-than-normal brain age estimation, which is reflected by a positive BPAD and most pronounced in the ADD group (Table 1, Fig. 2). BPAD values for SCD (*p* = 0.03), MCI (*p* < 0.001), and ADD (*p* < 0.001) were significantly different from zero, demonstrating an elevated brain age compared to their chronological age. In contrast, the HC group (*p* = 0.475) did not show a significant difference from zero, indicating that their BPAD values were not significantly elevated.

An additional analysis was performed to investigate the effect of scanner variability, more specifically multi-scanner vs. single-scanner results, by looking at the variable distribution of a subset of the dataset (*N* = 119), including patients with more stringent selection criteria (read. single scanner, single protocol, and single center). This resulted in narrower age (read: brain age, chronological age and BPAD) variable distributions for each diagnostic group with less overlap between the groups (Supplementary material 1).

#### The cross-sectional relationship between BPAD, brain age, chronological age and MMSE

**Fig. 3:**
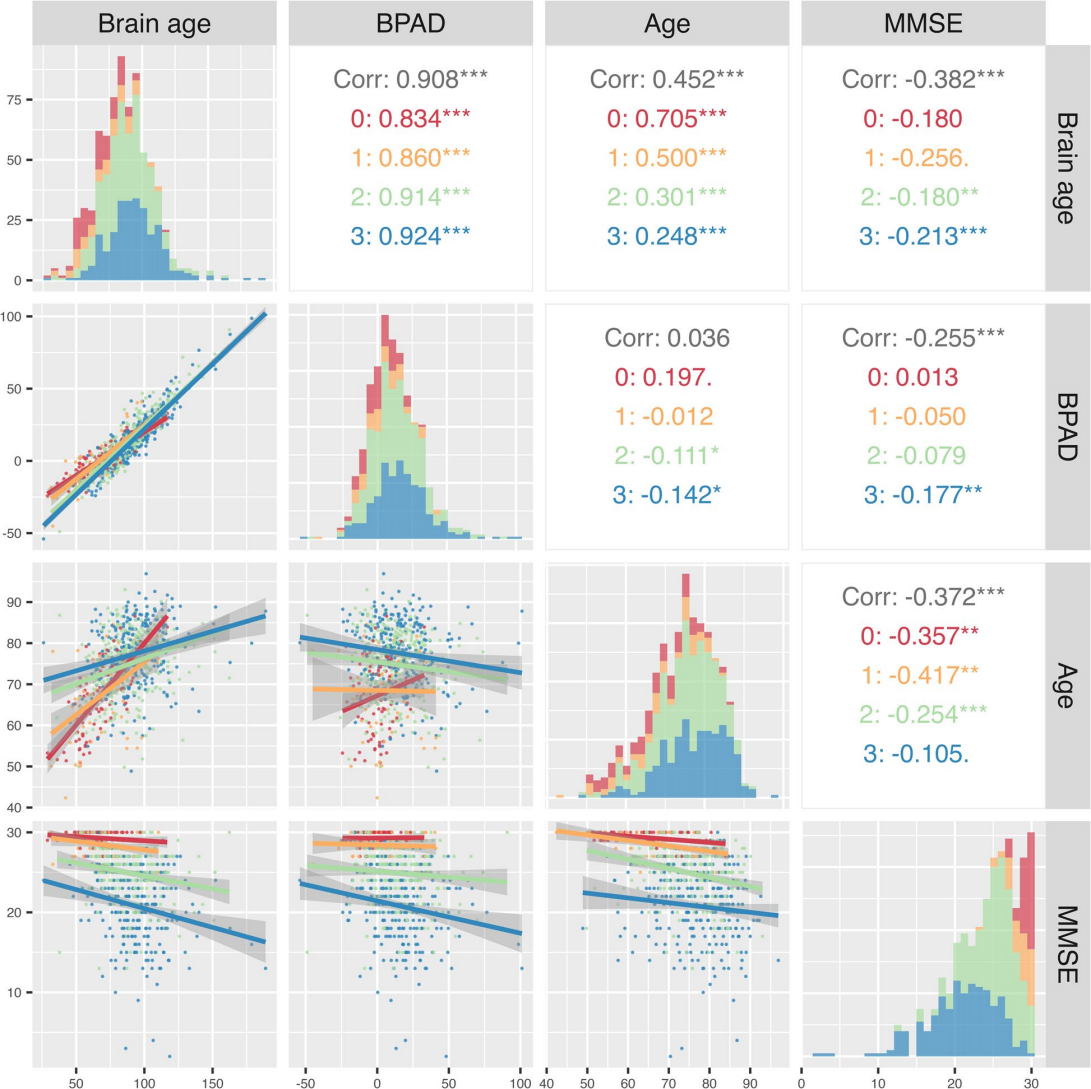
Correlation matrix. Correlogram of the following selected variables: Brain-predicted age difference (BPAD), brain age, Mini-Mental State Examination (MMSE) score and chronological age (Age). Figures are color-coded based on diagnostic index; healthy controls, HC (red), subjective cognitive decline, SCD (yellow), mild cognitive impairment, MCI (green), Alzheimer’s disease dementia, ADD (blue). Below diagonal: visualization of correlation graphs per variable combination. Diagonal: depiction of variable distribution. Above diagonal: correlation coefficients for (from top to bottom): total dataset (Corr), HC, SCD subjects, MCI patients, and ADD patients. *p < 0.05, **p < 0.01, ***p < 0.001

As part of the descriptive and exploratory analysis, a correlogram was constructed using a selection of variables. These include BPAD, brain age, chronological age and MMSE (HC = 71, SCD = 59, MCI = 302, ADD = 259) (Fig. 3). To illustrate the diagnostic subgroup distributions, the min–max ranges of the MMSE scores were reported: CN [27–30], SCD [23–30], MCI [13–30], and ADD [2–30]. Significant (positive) correlations for brain age to BPAD (*r* = 0.908, *p* < 0.001) and Age (*r* = 0.452, *p* < 0.001) were found, as well as inverse (negative) correlations with MMSE (meaning an increase in brain age is correlated to a decline in MMSE (*r* = -0.382, *p* < 0.001)). MMSE score was additionally significantly correlated to the following variables: BPAD (*r* = -0.254, *p* < 0.001) and Age (*r* = -0.373, *p* < 0.001).

#### Classification performance

The classification performance of BPAD, brain age, and chronological age varied across different diagnostic groups (Table 2). The highest AUC was observed in the comparison between ADD and HC, where brain age achieved an AUC of 0.858 ([CI: 0.817–0.900]), followed by chronological age (AUC = 0.808 [CI: 0.759–0.857]), and BPAD (AUC = 0.764 [CI: 0.759–0.858]), respectively. The lowest AUC was observed in the comparison between SCD and HC, where all three measures had AUC values ranging from 0.551 [CI: 0.456–0.645] to 0.577 [CI: 0.483–0.675]. Overall, brain age showed a trend in outperforming BPAD and chronological age in terms of diagnostic accuracy. Nevertheless, the reported *p*-values and overlapping confidence intervals indicated no significant difference.

**Table 2:** Classification performance of BPAD, Brain Age and Chronological age. Cognitively healthy controls; HC. Subjective cognitive decline subjects; SCD. Mild cognitive impairment patients; MCI. Alzheimer disease dementia patients; ADD. Brain predicted age difference; BPAD. Sensitivity; SENS. Specificity; SPEC. SENS and SPEC were computed at the cut-off determined by Youden’s index. Area under the curve; AUC. Confidence interval; CI. Highest AUC is marked in bold. ANOVA with *post-hoc* Tukey correction was conducted to compare the AUC values of the three different predictors (chronological age, BPAD, and brain age), as well as for each pairwise comparison between diagnostic groups. All *p*-values were non-significant, and thus have been combined into a single column representing the overall *p*-value

	**Chronological age**			**BPAD**			**Brain age**			
Diagnostic groups	**SPEC (%)**	**SENS (%)**	**AUC [CI 95%]**	**SPEC (%)**	**SENS (%)**	**AUC [CI 95%]**	**SPEC (%)**	**SENS (%)**	**AUC [CI 95%]**	***p***
SCD VS. HC	81.3	35.4	0.551 [0.456, 0.645]	84.6	36.9	0.567 [0.456, 0.645]	87.9	32.3	0.577 [0.483, 0.675]	NS
MCI VS. HC	81.3	54.5	0.743 [0.686, 0.800]	85.7	53.9	0.715 [0.662, 0.786]	89.0	56.1	0.797 [0.749, 0.846]	NS
ADD VS. HC	73.6	73.0	0.808 [0.759, 0.857]	85.7	60.3	0.764 [0.759, 0.858]	**89.0**	**73.0**	**0.858 [0.817, 0.900]**	NS
MCI VS. SCD	50.8	79.9	0.685 [0.613, 0.758]	52.3	70.2	0.636 [0.562, 0.711]	35.6	93.1	0.705 [0.634, 0.776]	NS
ADD VS. SCD	87.7	51.3	0.760 [0.698, 0.822]	81.5	49.4	0.681 [0.610, 0.753]	72.3	73.0	0.776 [0.713, 0.839]	NS
ADD VS. MCI	78.1	39.3	0.601 [0.544 0.647]	52.3	70.2	0.554 [0.507 0.601]	43.8	73.0	0.589 [0.543, 0.635]	NS

## Longitudinal evaluation of brain age and BPAD

### Brain age and BPAD increase over time

The longitudinal analysis was performed on a subset of the study population (*N* = 125) remaining in the same diagnostic group at BL and FU. This subset consisted of individuals with longitudinal data, containing MMSE scores for both BL and FU (time between BL and FU MMSE (mean ± SD): 1.9 ± 1.2 years) and MR images with BL and FU within 2 years (Time between BL and FU MRI (mean ± SD): 1.5 ± 1.1 years). Brain age and BPAD were not significantly different between BL and FU for any of the diagnostic groups, except for the brain age values of the controls (*p* = 0.037) (Table 3). To model differences in BPAD change between BL and FU for different diagnostic groups, linear mixed models were fitted as described in the method section. No significant differences in BPAD change were found between any of the pairwise comparisons of the different diagnostic groups.

**Table 3:** Longitudinal brain age increase over time. Data represented as mean ± standard deviation (SD) or percentages (where applicable)

Disease stage	Brain age, BL	Brain age, FU	Brain age, *p*-value	BPAD, BL	BPAD, FU	BPAD, *p*-value	MMSE, BL	MMSE, last FU	Time between BL and FU MRI (years)	Time between BL and last FU MMSE(years)	Increase in brain age (%)
HC (*n* = 15)	65.67 (14.32)	72.41 (17.41)	0.037*	-0.145 (10.79)	5.651 (12.82)	0.051	29 (2)	29 (3)	1.5 (1.7)	1.5 (1.0)	10.3
SCD (*n* = 17)	71.37 (17.60	74.28 (10.63)	0.330	3.59 (14.14)	4.93 (8.15)	0.649	27 (2)	27 (2)	1.5 (1.0)	2.0 (1.2)	4.08
MCI (*n* = 60)	86.69 (18.72)	86.63 (17.02)	0.976	14.31 (13.81)	12.77 (13.34)	0.407	26 (3)	24 (5)	1.5 (1.0)	2.0 (1.2)	-0.07
ADD (*n* = 33)	85.33 (24.23)	89.36 (24.76)	0.406	13.88 (22.72)	16.31 (22.42)	0.608	23 (3)	21 (5)	1.5 (1.0)	1.9 (1.3)	4.71
Total (*n* = 125)	81.72 (21.04)	83.96 (19.62)	0.174	11.01 (17.12)	11.78 (16.08)	0.631	26 (3)	24 (5)	1.5 (1.1)	1.9 (1.2)	1.49

Brain Predicted Age Difference; BPAD

*HC* Cognitively healthy controls, *SCD* Subjective cognitive decline subjects, *MCI* Mild cognitive impairment patients, *ADD* Alzheimer disease dementia patients, *BL* Baseline, *FU* Follow-up, *MMSE* Mini-Mental State Examination

*p < .05

### The relation between BPAD and cognitive evolution over time

The influence of BPAD on the cognitive evolution over time, expressed as the change on MMSE total score per year, was also investigated. Longitudinal data on baseline BPAD and cognitive evolution over time (*N* = 331), as well as data on annualized BPAD differences and cognitive evolution over time (*N* = 136), were collected. Results showed no significant Spearman correlation between the baseline BPAD and cognitive evolution over time, nor between the annualized BPAD difference and cognitive evolution over time for any of the diagnostic groups (Supplementary Material 2).

## The relation between BPAD and conversion

### Incidence of conversion

To investigate if a higher BPAD influences the incidence of conversion from MCI to ADD, logistic regression was performed on a subset (*N* = 323) of the complete dataset which contained information on conversion incidence and time-to-conversion. The subset contained MCI patients converting to ADD (*N* = 102) and subjects that did not convert to a later stage in the AD continuum (*N* = 221). BPAD was used as an independent variable, assuming a continuous increase of change from lowest to highest BPAD. No association for BPAD with the incidence of conversion from MCI to ADD was found (Table 4). Note that due to the small number of subjects (N < 10) in other combinations of diagnostic groups, these analyses have not been reported.

**Table 4:** Incidence of MCI to ADD conversion. Data is represented as AUC with 95% confidence interval [CI] or percentages (%)

Progression stage	Specificity (%)	Sensitivity (%)	AUC [CI 95%]
MCI to ADD (*N* = 102)	80.5	28.4	[0.462, 0.599]

MCI to ADD progression stage represents the positive class (102) versus total considered sample (*N* = 323)

*MCI *Mild cognitive impairment, *ADD* Alzheimer disease dementia patients

### Time to conversion

**Fig. 4:**
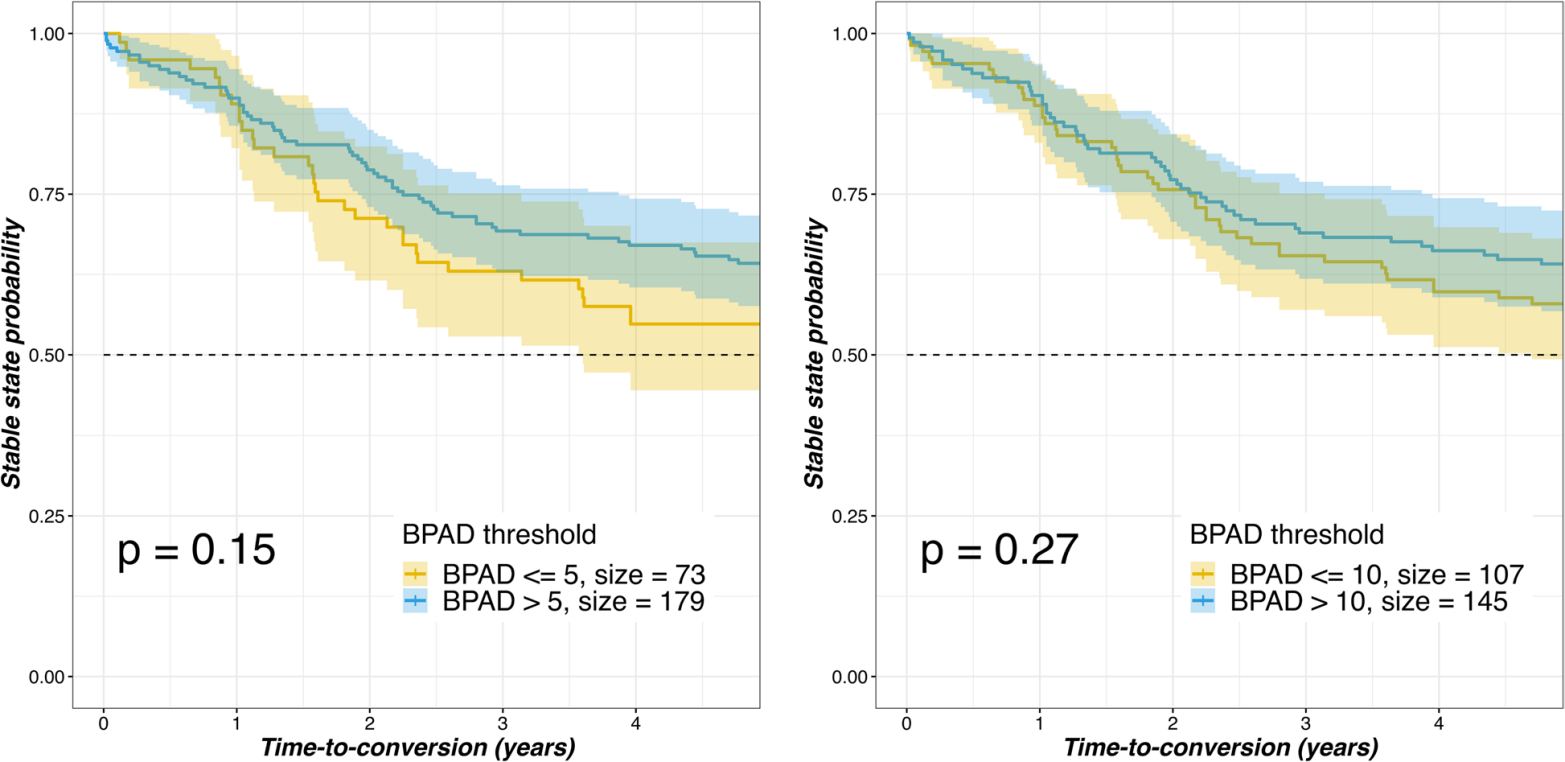
Kaplan–Meier plots stratified with BPAD thresholds—progressive vs non-progressive (stable-state) MCI. Brain predicted age difference; BPAD. Mild Cognitive Impairment; MCI

To investigate if a higher BPAD in later stages of the continuum influences conversion time, a survival analysis evaluating the incidence of conversion over time using different baseline BPAD thresholds (> 5, > 10, > 15, > 20, > 25, > 30) was performed for progressive vs. non-progressive MCI patients with, besides conversion incidence, additional data regarding the time to conversion (*N* = 252). The log-rank test showed there was a noticeable trend between the BPAD thresholds and the incidence of conversion for the > 5 and > 10 BPAD thresholds (Fig. 4). However, no significant differences were seen for any of the groups (not shown).

### The relation between BPAD and lifestyle factors

The relationship between BPAD and lifestyle factors, specifically smoking behavior, and alcohol usage, on BPAD was assessed as well. Collected data regarding smoking behavior (*N* = 378) was categorized in non-smokers (*N* = 247), smokers (min. 6 cigarettes/day) (*N* = 37) and ex-smokers (*N* = 94). Current smoking status ((mean ± SD) 14.71 ± 21.52), transition from smoking to non-smoking ((mean ± SD) 15.46 ± 18.48)), and non-smoking ((mean ± SD) 15.37 ± 19.65) did not have a significant impact on BPAD (*p* > 0.05). Data regarding alcohol usage (*N* = 370) was divided in non-/sporadic drinkers (*N* = 124), moderate drinkers (*N* = 207), and heavy drinkers (> 2 consumptions/day, *N* = 39). Drinking behavior was found to have a significant effect on BPAD, with pairwise comparisons revealing significant differences between non-/sporadic drinkers ((mean ± SD) 16.16 ± 18.46) and heavy drinkers ((mean ± SD) 26.30 ± 24.97) (*p* = 0.0135), as well as moderate drinkers ((mean ± SD) 15.39 ± 20.81), and heavy drinkers (*p* = 0.0396). No significant differences were seen for non-/sporadic drinkers vs. moderate drinkers (*p* > 0.05).

## Discussion

In this multicentric clinical study, the effects of brain age and BPAD on disease progression, conversion to other disease stages and lifestyle factors were investigated, to explore the potential value in routine clinical practice. The key novelty centers on several elements: firstly, to the validation of the novel brain age model on a large diverse multi-center clinical dataset, indicating readiness for routine practice; secondly, offering an easy interpretable alternative to existing complex models, making it more accessible to clinicians and researchers; thirdly, the model’s evaluation on a dataset comprising subjects in different cognitive stages across the entire AD continuum, including subjective cognitive decline (SCD), which, to our knowledge, has hardly been studied with respect to brain age [26]; and lastly, the confirmation of a significant association between brain age and certain lifestyle factors, despite considerable variability.

### Brain age across the AD spectrum

The study found that as individuals progressed along the AD continuum, their brain age increased more than their chronological age. This suggests a cumulative effect, that the brain ageing process in individuals with AD is accelerated, and this becomes more pronounced as the disease advances, which corresponds to prior research outcomes [5, 7, 21]. Beyond the sensitivity of the brain age model to pathology, the study demonstrates the generalizability of the brain age model, as it effectively applies to different groups of healthy controls with a comparable prediction error [21]. It should be noted that an MAE of 0 may not be desirable, as it could fail to account for inherent biological variability.

Furthermore, no significant effects were seen for higher BPAD in relation to conversion time from MCI to ADD, which is contradictory to earlier findings. However, the previously conducted studies were analyzed using ADNI data rather than in the context of a real-world clinical setting [15, 35, 36, 59].

### Brain age and cognitive performance

The results indicate that the correlation between MMSE and BPAD, along with brain age is predominantly evident in individuals with MCI and ADD, suggesting disease stage to drive the relationship. The MMSE vs. BPAD correlation is more meaningful across the entire sample (*r* = -0.254, *p* < 0.001), as the MMSE distribution is constrained within each group ([min–max range] HC [27–30], SCD [23–30], MCI [13–30], and ADD [2–30]). This might be a possible explanation for this observation, where especially the HC and SCD samples score high on the MMSE with a low variability. As individuals progress along the AD continuum, lower MMSE scores with greater variability are observed, likely attributed to disease-specific factors, rather than chronological aging, which has also been previously noted as a factor that may compromise its specificity for detecting cognitive impairment (also supported by the non-significant correlation between MMSE and chronological age in the ADD group).

In addition, the subgroup correlations are less negative than the overall correlation. Here, the potential impact of confounding factors, subgroup-specific variability (Simpson’s paradox [60]) and subgroup sizes on correlation patterns needs to be considered. This underscores the importance of investigating correlations not only in the entire dataset but also in subgroups individually to gain a comprehensive understanding of the data.

Lastly, it needs to be considered that the MMSE is known to have a tendency to demonstrate high ceiling effects, particularly in individuals with mild cognitive symptoms [61]. Consequently, it may not be the most optimal cognitive tool for correlation analysis in this context, especially considering that the majority of the study cohort consists of HC subjects, individuals with SCD, and MCI patients. Unfortunately, data on more suitable cognitive assessment tools, such as MoCA scores, were insufficiently available in this dataset to perform meaningful statistical analysis. Indeed, in Belgium, the National Institute for Health and Disability Insurance requires the MMSE for reimbursement of symptomatic pharmacological treatments for AD, which is why the MMSE is still commonly used in our memory clinics.

Notably, the findings reveal a correlation between brain ageing in AD and overall cognitive health. However, no significant Spearman correlation between BPAD and cognitive state over time was found, possibly due to the fact that the MMSE measure is an initial cognitive screening tool, influenced by factors such as depression and the individual’s state of mind at the time of testing.

### Brain age and lifestyle

When exploring the clinical applications of BPAD, BPAD may have potential as an endpoint for evaluating the effects of lifestyle interventions on accelerated ageing processes. Furthermore, BPAD was significantly different between non-sporadic and heavy drinkers, which was also reported in previously published literature, [7, 62], suggesting that (heavy) alcohol consumption has implications for brain health and ageing. It is worth noting that this observation does not establish a causal relationship, but it does suggest that certain lifestyle choices can potentially influence brain ageing trajectories, highlighting the importance of considering modifiable risk factors in relation to brain health.

### Brain ageing across the lifespan

The study also reported instances of very high brain ages surpassing the conventional human lifespan, which suggest significant neurodegeneration. Although this might partly be due to the linear nature of the brain age model, it could also be attributed to the clinical nature and diversity of the dataset. For example, variations in MRI scan quality or unflagged artifacts might contribute to less accurate segmentation during image processing, which in turn can be attributed to the elevated (and decreased) brain age predictions.

Correlation strengths between chronological age and both BPAD and brain age were not uniform across different cognitive states, emphasizing the complex, often not-linear, interactions between brain changes and age. However, there were notable differences in the classification performance between brain age, BPAD, and chronological age, where brain age generally showed the highest AUC values and better classification performance across the different diagnostic groups. In addition, the observation of instances where BPAD exhibited a lower AUC value than chronological age does not necessarily mean it is less effective in capturing pathological deviations beyond chronological ageing, e.g. age bias might be contributing to this inconsistency. Nevertheless, it was seen in each metric (brain age, BPAD and chronological age) that when the cognitive states diverge more widely, the AUC values tend to increase, suggesting improved differentiation between cognitive conditions. However, this should be interpreted with caution since, despite the importance of the AUC as a criterion for discriminative power, areas under two ROC curves can be the same although the curves are considerably different, indicating that *p*-values evaluating differences between AUC values are difficult to interpret [63]. It is imperative to acknowledge the intricate interplay between brain age estimates, chronological age, and BPAD in delineating cognitive conditions across a heterogeneous cohort.

Intriguingly, longitudinal brain age analyses revealed no significant changes within the diagnostic groups except for the control group, where a noteworthy increase was observed (*p* = 0.037), which, apart from the small sample size (*N* = 15), prompts consideration of various unmeasured confounders that may have influenced the observed differences.

### Limitations

It is important to note that the temporal distribution of follow-up times throughout the study lacked uniformity. This consequential variability in follow-up intervals presents a challenge in accurately determining the duration between conversion events and clinic visits. This limitation underscores the importance of implementing more standardized follow-up protocols in future studies to enhance our understanding of the temporal dynamics of disease progression. Nevertheless, this variability is inherent to the nature of routine clinical practice, given the heterogeneity in symptom representation and disease progression rates associated with AD, as it also largely determines the frequency and timing of follow-up visits in clinic.

Accordingly, the potential impact of a number of non-measured confounders, including demographic factors (e.g. ethnicity) [12], psychological factors (e.g. mental health conditions, stress levels, and emotional well-being), other lifestyle factors (e.g. substance/medication use, physical exercise, diet, sleep patterns, comorbidities) and environmental factors (e.g. socio-economic status, exposure to toxins and pollutants and access to healthcare resources) [16] can been seen as a limitation of the current study. Their potential contribution to accelerated ageing, together with the presence of genetic predisposition, reflects the complexity to accommodate for individual ageing trajectories. Determining the weight and relative contribution of each individual variable to brain ageing in further studies is imperative. The same reasoning applies to the definition of “cognitively healthy”, which in this study was based on neuropsychological test performance, while other non-measured confounders might also be relevant in this regard. This might, however, be considered a general concern in brain age research, reflecting the importance of providing a comprehensive definition of a (cognitively) “healthy” brain.

In addition, the brain age model that was used only captures linear relationships, which may oversimplify the complexities observed in ageing, as noted in the study by Bethlehem et al. in 2022 [5]. Furthermore, the model tends to overestimate the age of younger subjects and underestimate the age of older subjects, indicating the need to statistically correct for chronological age bias in brain age estimation models, and might also be the reason why the correlation between chronological age and brain age was lower than the correlation between chronological age and BPAD. However, analyses involving age corrected BPAD as an outcome variable need to be interpreted with caution, since age is comprised in BPAD. When correcting for age, specific contribution of age to BPAD may be diminished or removed, potentially impacting the interpretation of the findings. Regarding the predictive value of brain age in the conversion from SCD/MCI to ADD, it is important to acknowledge its potential limitations compared to other techniques such as Aβ and tau protein assays in CSF and advanced neuroimaging methods like amyloid- and tau-PET. However, besides its non-invasive nature and widespread availability, it offers direct insights into individual brain ageing and the structural alterations linked to AD pathology, rendering it a valuable complementary metric [33]. Nonetheless, even though brain age research has initially mainly been focusing on structural MRI, the incorporation of various neuroimaging modalities may improve the sensitivity of BPAD in e.g. preclinical AD detection [64, 65], as alternative studies have effectively developed brain age models using various other methods, including diffusion MRI [66–68], functional connectivity [69–74], and metabolic PET [27, 75].

Furthermore, we acknowledge that the difference in age distributions between clinical cohorts is a limitation in this study. Since there is a non-linear dependency of brain age on age in the linear brain age model, which was not corrected, this might partially explain the observed differences in BPAD. This non-linear dependency results in the intrinsic overestimation of age (positive BPAD) in the cognitively healthy sample of this paper. A correction approach has, however, been suggested by Smith et al. 2019 [76]. Besides chronological age bias, the strength of the findings can also be hampered by variability in the MRI data.

Moreover, the reproducibility of volumetric measurements across different centers was not directly verified in this study due to the nature of the dataset, which consisted of scans performed as part of routine clinical practice. However, reproducibility testing for the software’s automated volumetric measurements using three different MRI scanners was previously conducted and verified [77]. Nevertheless, a subgroup analysis on MRI data from a single site using a single scanner with uniform acquisition protocol showed stronger differences in brain age between diagnostic groups (Supplementary results) than for the complete multi-site cohort. This underscores an important limitation inherent to using neuroimaging; multi-site variability effects [59]. As a side note, an anticipated, though not to be diminished, confounding factor, is the systematic evaluation of motion-related artifacts significantly altering brain age estimates [78]*.* Thus, more diverse cohorts and longer-term longitudinal studies that include reproducibility testing between centers, multi-modal imaging data, and additional tests for cognitive assessment (beyond MMSE) are needed to investigate the potential added value of brain age and BPAD as outcome measures to test the effectiveness of lifestyle changes / interventions on brain health and prevention of dementia. At the same time, the majority of studies have concentrated on analyzing brain age trajectories at a group level; investigating individual case studies, including longitudinal and standardized repeated MRI assessments, can identify unique patterns that may not be evident in group-level analyses. Lastly, the “early system-integrity" perspective of cognitive and biological aging, that posits that individuals exhibit varying levels of brain and body health starting from early in life, should also be taken into consideration when evaluating the rate and trajectory of the brain ageing process [65]. Future research could hereby further increase our understanding of the dynamics of brain age and BPAD in pathological ageing.

## Conclusion

In conclusion, the study highlights the potential of brain age and BPAD as objective measures for assessing pathological ageing. These metrics offer insights into disease progression, stage differentiation, and new approaches to the potential evaluation of (lifestyle) interventions. Ultimately, a comprehensive understanding of brain age and its implications in pathological ageing, as seen in AD, could aid in early detection and risk stratification, and guide the development of targeted interventions for individuals at risk of cognitive decline.

### Supplementary Information

Supplementary Material 1.

Supplementary Material 2.

## Data Availability

The datasets used and/or analyzed during the current study are available from the corresponding author on reasonable request.

## Abbreviations

Aβ: Amyloid-beta
ADD: Alzheimer's disease dementia
AD: Alzheimer's disease
AUC: Area Under the Curve
BL: Baseline
BPAD: Brain Predicted Age Difference
CSF: Cerebrospinal Fluid
FC: Frontal Cortex
FU: Follow-Up
GM: Gray Matter
HC: Cognitively healthy controls
HIP: Hippocampus
MAE: Mean absolute error
MCI: Mild cognitive impairment
MNI: Montreal Neurological Institute
MMSE: Mini-Mental State Examination
MRI: Magnetic Resonance Imaging
NIA-AA: National Institute on Aging and Alzheimer's Association
OC: Occipital Cortex
PC: Parietal Cortex
ROC: Receiver Operating Characteristic
SCD: Subjective cognitive decline
SD: Standard Deviation
TC: Temporal Cortex
WM: White Matter
