# Supplementary material for: Brain age as a biomarker for pathological versus healthy ageing – a REMEMBER study
Source: Alzheimers Res Ther. 2024 Jun 14;16:128. doi: 10.1186/s13195-024-01491-y (PMC11179390; doi:10.1186/s13195-024-01491-y)
Supplement: Supplementary file 1 — Supplementary Material 1. [file 13195_2024_1491_MOESM1_ESM.docx]

**
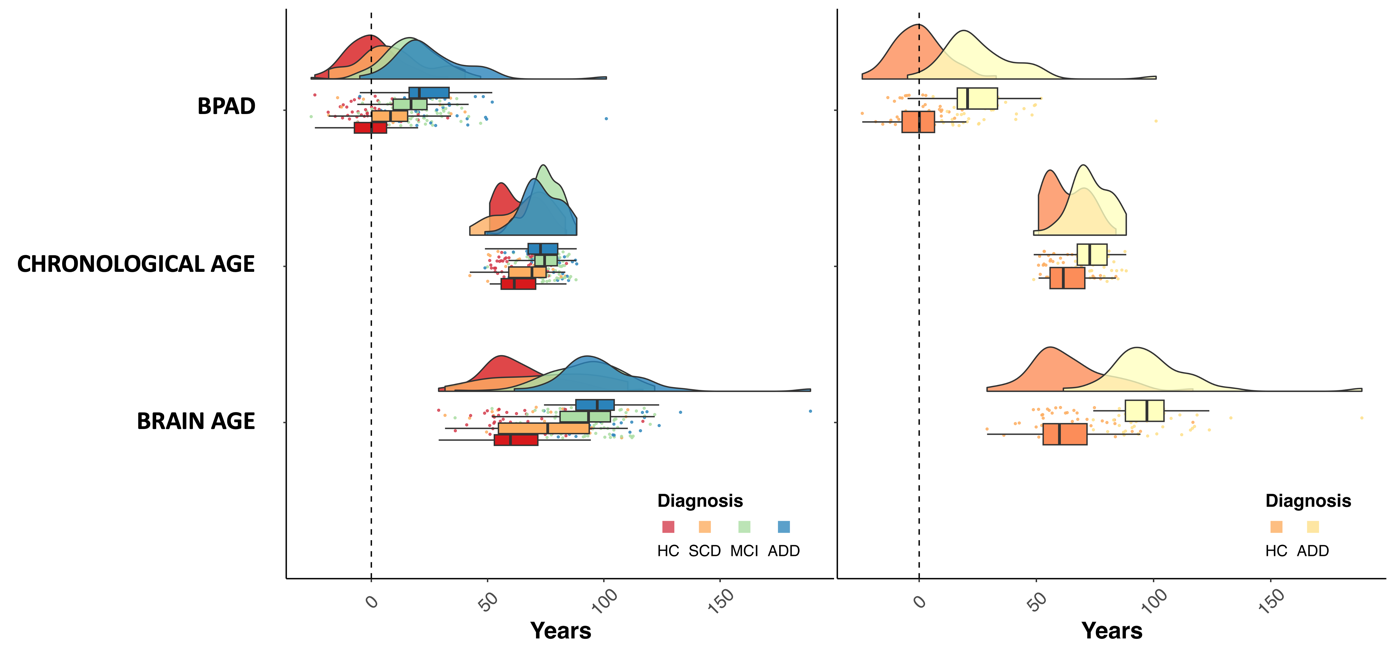
**

**Supplementary Material 1: Distribution of age variables per diagnostic group – single scanner, single protocol, single center**. **A)** All diagnostic groups B) HC and ADD. Cognitively healthy controls; **HC**. Subjective cognitive decline subjects; **SCD**. Mild cognitive impairment patients; **MCI**. Alzheimer disease dementia patients; **ADD.** Brain predicted age difference; **BPAD**. Age at baseline; **Chronological ageL**. Left: All diagnostic groups are visualized (HC in red, SCD in orange, MCI in green, and ADD in blue). Right: Only HC (orange) and ADD (yellow) groups are visualized.
