# Supplementary material for: Brain age as a biomarker for pathological versus healthy ageing – a REMEMBER study
Source: Alzheimers Res Ther. 2024 Jun 14;16:128. doi: 10.1186/s13195-024-01491-y (PMC11179390; doi:10.1186/s13195-024-01491-y)
Supplement: Supplementary file 2 — Supplementary Material 2. [file 13195_2024_1491_MOESM2_ESM.docx]

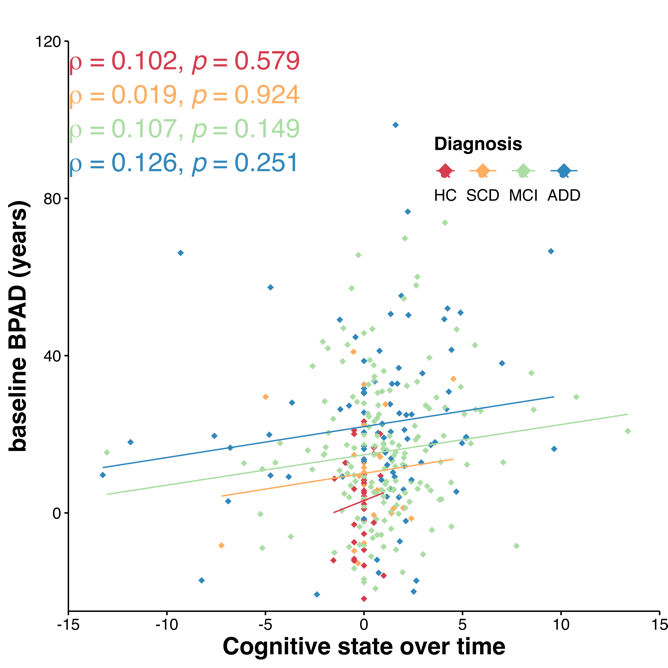

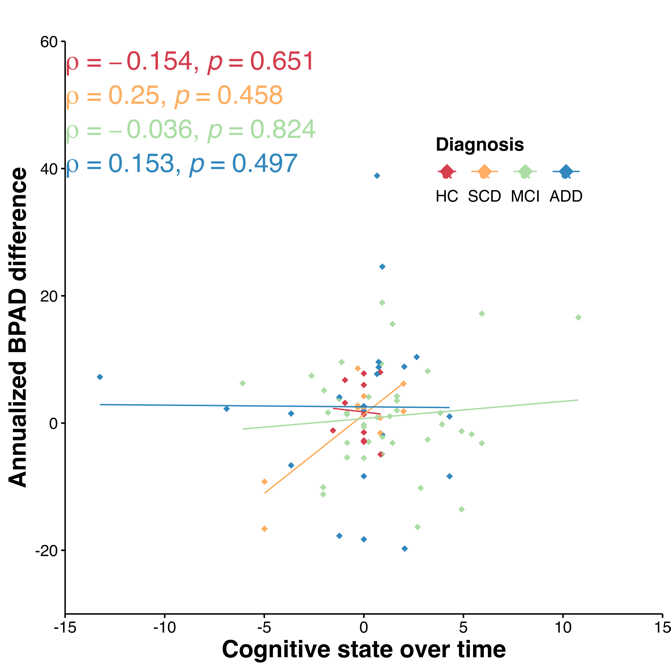


**Supplementary Material 2: BPAD and cognitive state over time**. **left)** baseline BPAD vs. cognitive state over time **right)** Annualized BPAD difference vs. cognitive state over time. Cognitively healthy controls, HC. Subjective cognitive decline subjects; **SCD**. Mild cognitive impairment patients; **MCI**. Alzheimer disease dementia patients; **ADD.** Brain predicted age difference; **BPAD**. The cognitive state over time (read. yearly difference in Mini Mental State Examination (MMSE)) was calculated by subtracting the follow-up (FU) MMSE score from the baseline (BL) MMSE and dividing it by the time between BL and FU in years. All diagnostic groups are visualized (HC in red, SCD in orange, MCI in green, and ADD in blue).
